# Effects of Chitosan-Coated Microdiet on Dietary Physical Properties, Growth Performance, Digestive Enzyme Activities, Antioxidant Capacity, and Inflammation Response of Large Yellow Croaker (*Larimichthys crocea*) Larvae

**DOI:** 10.1155/2022/4355182

**Published:** 2022-09-05

**Authors:** Jiahui Liu, Wenxuan Xu, Yongtao Liu, Yuntao Wang, Jianmin Zhang, Zhen Wang, Kangsen Mai, Qinghui Ai

**Affiliations:** ^1^Key Laboratory of Aquaculture Nutrition and Feed (Ministry of Agriculture and Rural Affairs), Key Laboratory of Mariculture (Ministry of Education), Ocean University of China, 5 Yushan Road, Qingdao, Shandong 266003, China; ^2^Laboratory for Marine Fisheries Science and Food Production Processes, Qingdao National Laboratory for Marine Science and Technology, 1 Wenhai Road, Qingdao, Shandong 266237, China

## Abstract

A 30-day feeding trial was designed to investigate the physical properties of chitosan-coated microdiet (CCD) and the effect of CCD on survival, growth performance, activities of digestive enzymes, intestinal development, antioxidant capacity, and inflammatory response of large yellow croaker larvae (initial weight: 3.81 ± 0.20 mg). Four isonitrogenous (50% crude protein) and isolipidic (20% crude lipid) microdiets were prepared with different concentrations of chitosan wall material by spray drying method (0.00%, 0.30%, 0.60%, and 0.90%, weight (chitosan) : volume (acetic acid)). Results showed that the lipid encapsulation efficiency (control: 60.52%, Diet1: 84.63%, Diet2: 88.06%, Diet3: 88.65%) and nitrogen retention efficiency (control: 63.76%, Diet1: 76.14%, Diet2: 79.52%, Diet3: 84.68%) correlated positively with the concentration of wall material (*P* < 0.05). Furthermore, the loss rate of CCD was significantly lower than the uncoated diet. Larvae fed the diet with 0.60% CCD had significantly higher specific growth rate (13.52 and 9.95%/day) and survival rate (14.73 and 12.58%) compared to the control group (*P* < 0.05). Larvae fed the diet with 0.30% CCD had significantly higher trypsin activity in pancreatic segments than the control group (4.47 and 3.05 U/mg protein) (*P* < 0.05). Larvae fed the diet with 0.60% CCD had significantly higher activity of leucine aminopeptidase (7.29 and 4.77 mU/mg protein) and alkaline phosphatase (83.37 and 46.09 U/mg protein) in the brush border membrane than those of the control group (*P* < 0.05). The intestinal epithelial proliferation- and differentiation-related factors (*zo-1*, *zo-2*, and *pcna*) in larvae fed the diet with 0.30% CCD had higher expression than those of the control group (*P* < 0.05). When the concentration of wall material reached 0.90%, the larvae had significantly higher superoxide dismutase activity than that of the control group (27.27 and 13.72 U/mg protein) (*P* < 0.05). Meanwhile, malondialdehyde contents were significantly lower in larvae fed the diet with 0.90% CCD than that of the control group (8.79 and 6.79 nmol/mg protein) (*P* < 0.05). 0.30%~0.60% CCD significantly increased the activity of total nitric oxide synthase (2.31, 2.60, and 2.05 mU/mg protein) and inducible nitric oxide synthase (1.91, 2.01, and 1.63 mU/mg protein) and had significantly higher transcriptional levels of inflammatory factor genes (*il-1β*, *tnf-α*, and *il-6*) than those of the control group (*P* < 0.05). The results indicated chitosan-coated microdiet had great potential in feeding large yellow croaker larvae in addition to reducing nutrition loss.

## 1. Introduction

At early stage of marine fish, high request of nutrition levels put forward more superior request to feed [1]. For a long time, live feeds have been used as initial feeding for larvae of aquaculture animals while they are expensive, unreliable, and prone to carry pathogenic bacteria [2]. Therefore, the aquatic feed industry paid more attention to artificial microdiets (MD) such as microbound diet (MBD) and microcoated diet (MCD) because of its balanced nutrition, good palatability, and so on. MBD has long been used at the early stage of aquatic animals [3]. However, the dissolution of water-soluble substances such as amino acids, vitamins, and minerals has always been the bottleneck of the development of MBD [4]. MCD can greatly reduce nutrient loss and improve water quality due to the protection of wall material compared to MBD. In recent years, some experiments proved that MCD were possible to partially replace or even completely replace live feed, and cellulose and gelatin have been widely applied as wall material [2, 4–7]. Suitable wall material to improve the processing technology of aquatic feed was of great significance.

Chitosan is synthesized by deacetylation of chitin which exists broadly in nature. Chitosan is almost insoluble in water and widely applied in the field of biology [8]. Chitosan-coated diets can improve water quality, reduce nutrient loss of feed, and enhance the immunity of cultured animals [9, 10]. Meanwhile, a great number of studies on animals have proved that chitosan can promote growth and inhibit bacteria [11–13]. Furthermore, chitosan has been fully affirmed as a drug coating and food cling film [14]. In the previous study, chitosan-FS (fluconazole-loaded solid lipid nanoparticles)-films might be effective as a neoteric drug administration for treating candidiasis via oral mucosa [15]. It can be seen that the application prospects of chitosan in the aquatic feed field are not only embodied in the physical properties of feed but also reflected in its excellent biological function. However, little information on the effect and appropriate proportion of chitosan as a coating material on aquatic animals was reported.

Large yellow croaker occupies an important position of marine culture fishes in China [16]. Chitosan-coated mirodiet might provide an optimal way to substitute live food and improve large yellow croaker larvae feeding. Therefore, this study was conducted to investigate the physical properties of CCD and the effects of CCD on survival, growth, antioxidant capacity, and inflammatory response of large yellow croaker larvae.

## 2. Materials and Methods

### 2.1. Preparation of Microcoated Diets

MBD was prepared as a precursor of microcoated diets (Table 1). Chitosan was purchased from Solarbio Biotechnology Co., Ltd. (Beijing, China), and the deacetylation degree was more than 90%. Chitosan coating solution were prepared in 2% glacial acetic acid at first. Then, the wall material was sprayed on the surface of the core, and the feed product was obtained after drying at 50 degrees. The process index of the coating machine was 12 m^3^/h air volume, 100 rpm rotary speed, 54.0°C air inlet temperature, and 0.20 sec pulse blowing time with 3.0 sec interval time. The fish were fed with MCD which processed with chitosan coating solution (0.30% chitosan, 0.60% chitosan, and 0.90% chitosan) [17, 18]. All preparation was done at the Nankou Base of Chinese Academy of Agricultural Science (Beijing, China).

### 2.2. Experimental Setup and Fish Rearing Conditions

Large yellow croaker larvae were purchased and raised in the Institute of Marine and Fisheries Research of Ningbo, China. Large yellow croaker larvae were fed with rotifers (*Brachionus plicatilis*) (0.5 × 10^4^ ~ 1.5 × 10^4^ individual L^−1^) from 3 to 7 days after hatching (DAH), fed with brine shrimp (*Artemia nauplii*) (1.0 × 10^3^ ~ 1.5 × 10^3^ individual L^−1^) from 5 to 10 DAH, fed with copepods (*Calanus sinicus*) from 8 to 14 DAH, and fed with the same MD from 12 to 14 DAH. After 15 days, the experimental diet was completely fed. The initial weight of large yellow croaker (3.81 ± 0.20 mg) was determined. Then, they were randomly divided into four groups with three replicates which culture 2000 fish in a blue storage tank about 150 L in each replicate. The control group was fed with uncoated microdiet, and the rest of the groups were fed with 0.30%, 0.60%, and 0.90% CCD, respectively.

Feed 7 times daily (6:30, 8:30, 10:30, 13:30, 15:30, 17:30, and 23:00). The feeding experiment was conducted for 30 days (pH value: 7.8~8.2, salinity: 21‰~24‰, temperature: 23~26°C). Keep the cycle of 12 h of light each day. To keep clear and healthy water throughout the experiment, two-thirds of water in the bucket was replaced daily with fresh seawater to suck away feces and uneaten food.

### 2.3. Sample

After starvation for 24 h, larvae were sampled. 40 larvae were collected from each barrel randomly and dissected to obtain the visceral mass (VM) for measuring enzyme activity and gene expression assays, which contains the mixture of the heart, spleen, pancreas, liver, and intestine. Fifty larvae were randomly selected to separate pancreatic segments (PS) and intestinal segments (IS) under an anatomical microscope at 0°C. To determine the crude protein, crude fat, and moisture, the remaining larvae from each container were gathered.

### 2.4. Analytical Methods

#### 2.4.1. Morphology of Microcoated Diets

A scanning electron microscope was used to observe the surface morphology of CCD (3rd Generation of VEGA SEMs, Tescan, Czechia). The preparation method of the sample was putting the microspheres on the microscope sample holder and sputtered gold in argon atmosphere to obtain a uniform gold coating of the microspheres [9]. The aim of this analysis was to visualize the surface morphology of the microcoated diets after one month of storage.

#### 2.4.2. Setting Velocity

Soak several pellets in water for 1 min in advance and fill a measuring cylinder with ultrapure water (6 cm in diameter and 1 L in volume). Then, put the pellet under the surface of the water column with a straw, and record the time it takes for the pellet to sink to 20 cm with the accuracy of 0.01 s. Repeat 30 times to reduce the error.

#### 2.4.3. Lipid Encapsulation Efficiency (LEE) and Nitrogen Retention Efficiency (NRE)

The method was referred to a previous study [19]. LEE was measured three times as follows: a microcoated sample of 1 g was quickly rinsed with 50 mL ether, and LEE was the weight of lipid substances in the feed sample after flushing.

Triplicate samples of the feeds (1 g) were immersed in 100 mL 35% NaCl solution at 20°C for 60 min. The feed samples were tested for NRE after filtration and drying [19].

#### 2.4.4. Leaching Rate

Firstly, weight out three feed samples (10 g), and one (control group) was baked in the oven at 105°C until constant weight. Two samples (test group) were placed in a wire screen in a container (5.5 cm deep) with seawater (15 g·L^−1^ salinity, pH 8.0) for a parallel test. After soaking for 30, 60, 90, and 120 min, screen was lifted up and down three times from the bottom to the surface of water. The feed in the screen was placed in a 105°C oven and baked to constant weight. All determinations were made in triplicate.

#### 2.4.5. Component Analysis

Samples of MBD, MCD, and larvae were dried to constant weight in an oven at 105°C for determination. Using Kjeldahl method (Kjeltec TM 8400, FOSS, Tecator, Sweden) and multiplying nitrogen by 6.25 to estimate crude protein. Soxhlet method (B-801, Switzerland) was applied to determine the crude lipid. Each sample was analyzed three times.

#### 2.4.6. Enzyme Activity Assay

About 0.1 g of IS and PS was ground with 1 mL phosphate-buffered saline (4°C, pH = 7.4) and centrifuged at 3000 g for 10 min. The supernatant was collected for the determination of *α*-amylase, trypsin, and lipase. Following the method of Crane et al. [20], the purified brush border membranes (BBM) of the IS were extracted for the measurement of alkaline phosphatase activity (AKP). According to previously described methods [21], leucine-aminopeptidase (LAP) was estimated.

About 0.1 g of VM were ground with 1 mL normal saline (0°C, pH = 7.4) and centrifuged at 3000 g for 10 min. The supernatant was extracted for the determination of superoxide dismutase (SOD), total antioxidant capacity (T-AOC), catalase (CAT), the content of malondialdehyde (MDA), the content of glutathione (GSH), peroxidase (POD), lysozyme (LZM), total nitric oxide synthase (TNOS), inducible nitric oxide synthase (iNOS), and constitutive citric oxide synthase (cNOS). All detection kits were purchased from the Nanjing Jiancheng Institute of Biological Engineering, China.

#### 2.4.7. RNA Extraction and Real-Time Quantitative Polymerase Chain Reaction (RT-qPCR)

About 0.01 g of VM were added into 1 mL Trizol (Takara, Japan) reagent for grounding to get homogenate. After the homogenate were extracted and purified, the total RNA was detected for integrity and then assessed by a Nano Drop®2000 spectrophotometer (Thermo Fisher Scientific, USA) to test the concentration. Then, cDNA was reverse transcribed of RNA by Prime Script-RT reagent Kit (Takara, Japan). Use a quantitative thermal cycler (CFX96TM Real-Time System, BIO-RAD, USA) to carry out real-time quantitative polymerase chain reaction [22]. PCR primer sequences used in this study were directly synthesized (Table 2).

### 2.5. Calculation and Statistical Methods

#### 2.5.1. Growth Performance

(1) $$\begin{array}{c} \text{Survival rate }\left(\text{SR}\right)\left(\%\right)=\frac{N_{t}\times 100}{N_{0}},\\ \text{Specific growth rate }\left(\text{SGR},\%{\text{day}}^{-1}\right)=\frac{\left(\ln W_{t}-\ln W_{0}\right)\times 100}{D}. \end{array}$$

*N*_*t*_:Total number of larvae at the ending of experiment

*N*_0_: Total number of larvae at the beginning of experiment

*W*_*t*_: Final body weight of larvae

*W*_0_: Initial body weight of larvae

*D*: Total number of experimental days.

#### 2.5.2. LEE and NRE

(2) $$\begin{array}{c} \text{LEE }\left(\%\right)=\left(\frac{1-\text{weight of lipid on surface}}{\text{total lipid weight}}\right)\times 100,\\ \text{NRE }\left(\%\right)=\left(\frac{\text{weight of protein after drying}}{\text{the total weight of protein}}\right)\times 100. \end{array}$$

#### 2.5.3. Leaching Rate

The stability of CCD in seawater is expressed by the leaching rate which is calculated as follows.

(3) $$\begin{array}{c} C=\left(m_{0}-m\right)\times \frac{100}{m_{0}}. \end{array}$$

*C*:Leaching rate, %

*m*_0_:The weight of control group after drying, unit is g

*m*: The weight of test group after drying, unit is g.

#### 2.5.4. Statistical Analyses

Data in this study were analyzed by one-way analysis of variance (ANOVA) using SPSS Statistics for mac V26.0 and then determined by Tukey's range test. The significance level was determined as *P* < 0.05, and results were exhibited as mean ± S.D. (standard deviation) and ±S.E.M. (standard error of means).

## 3. Results

### 3.1. Physical Properties of CCD

#### 3.1.1. Scanning Electron Micrograph

**Figure 1:**
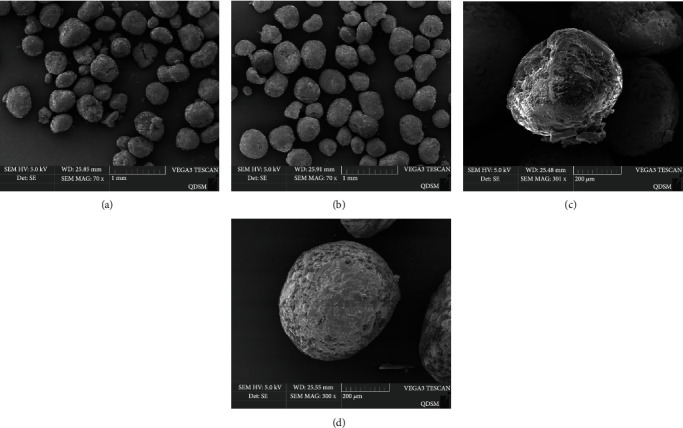
Microphotographs of the chitosan-coated diets before coating (a, b) and after coating (c, d).

Microphotographs showed the configuration of feed microdiets before (a, b) and after (c, d) coating processing. The shape of the CCD remained intact, with less feed crumbs and a relatively ellipse or spherical shape (Figure 1(c)). A complete and continuous film could be seen on the surface of the core. Micrograph showed many cracks in the structure of uncoated feed as well as a lot of fragments of the feed after a month of storage (Figure 1(a)).

There were only a few oil droplets on the surface of CCD, which explained the reason chitosan coating could significantly improve LEE (Figure 1(d)). However, after one month of storage, more oil seeped out of the pore of the uncoated MD (Figure 1(b)).

#### 3.1.2. Setting Velocity, LEE, NRE, and Leaching Rate

Setting velocity of CCD exhibited a trend of decrease while no significant difference was found among treatments (*P* > 0.05) (Table 3). Both lipid encapsulation efficiency (LEE) and nitrogen retention efficiency (NRE) significantly increased with increasing mass volume ratio of chitosan (*P* < 0.05) (Table 3).

**Figure 2:**
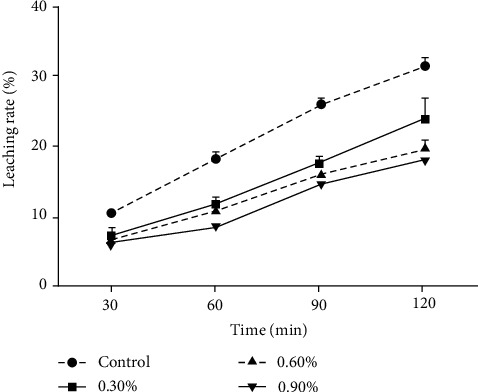
Leaching rate of total nutrients from microcoated groups and the control group (Means ± S.E.M., *n* = 3).

The leaching rate of different groups increased over time and significant differences was observed between MBD and MCD (Figure 2) (*P* < 0.05). 0.30% CCD exhibited 7.43 ± 0.60% loss of total nutrients during the first hour and then increased to 24.07 ± 1.86% which was significantly lower than MBD (*P* < 0.05) (Figure 2).

### 3.2. Effects of Dietary CCD on Large Yellow Croaker Larvae

#### 3.2.1. Survival Rate, Growth Performance, and Body Composition

The SR of larvae fed with 0.60% CCD was significantly higher than the control group (*P* < 0.05) (Table 4). Larvae fed diets with 0.60% and 0.90% CCD appeared higher FBW and FBL than the control group (*P* < 0.05) (Table 4). Meanwhile, SGR was significant higher in fish fed diets with CCD than the control group (*P* < 0.05) (Table 4). There were no significant differences in protein, lipid, and moisture among different groups of larvae in body composition (*P* > 0.05) (Table 5).

#### 3.2.2. Digestive Enzyme Activity

The activity of *α*-amylase was higher in IS of larvae fed with 0.06% CCD than the control group (*P* < 0.05) (Table 6). The activity of trypsin in IS of larvae fed with 0.30% chitosan coating diet had a significant increase compared with the control group (*P* < 0.05) (Table 6). However, the activity of lipase in larval IS and PS had no significant difference among different diets (*P* > 0.05) (Table 6).

With the concentration of chitosan increased, the activity of AKP and LAP in BBM increased at first and then decreased. Meanwhile, the activities of AKP and LAP in larval BBM was higher in larvae fed with 0.60% CCD than the control group (*P* < 0.05) (Table 6). However, the activity of LAP of larvae fed with 0.90% CCD decreased significantly compared to the control group (*P* < 0.05) (Table 6).

#### 3.2.3. Expression of Intestinal Development-Related Genes

**Figure 3:**
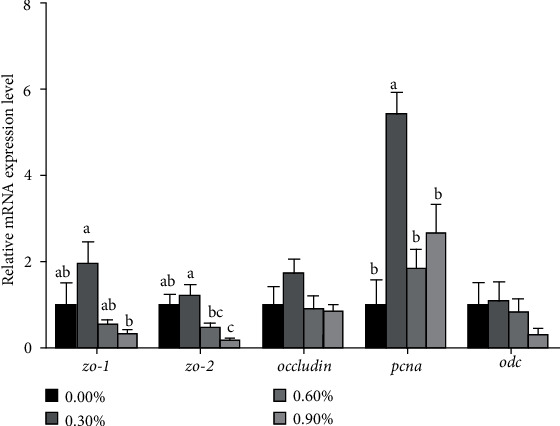
Effects of dietary CCD on *zo-1*, *zo-2*, *occludin*, *pcna*, and *odc* mRNA expression in a visceral mass. Vertical bars represent standard errors. There was no significant difference in bars bearing the same letters (*P* > 0.05, Tukey's test). *zo-1*: tight junction protein-1; *zo-2*: tight junction protein-2; *pcna*: proliferating cell nuclear antigen; *odc*: ornithine decarboxylase.

Larvae fed the diet with 0.30% CCD had significantly higher mRNA expression of *zo-1*, *zo-2*, and *pcna* in the IS than the control group (*P* < 0.05) (Figure 3). However, larvae fed with 0.90% CCD significantly decreased the mRNA expression of *zo-2* compared with the control group (*P* < 0.05) (Figure 3). No significant difference was observed in the mRNA expression of *occludin* and *odc* (*P* > 0.05) (Figure 3).

#### 3.2.4. Antioxidant Parameters

The activity of SOD in larvae fed the diet with 0.90% CCD was significantly higher than that in the control group (*P* < 0.05) (Table 7). The activity of T-AOC and CAT both showed a tendency to increase and then decrease (*P* > 0.05) (Table 7). However, the content of GSH was higher in larvae fed the diet with 0.60% CCD than the control group (*P* < 0.05) (Table 7). The content of MDA decreased with the increasing concentration of chitosan (*P* < 0.05) (Table 7).

#### 3.2.5. Inflammatory Response

**Figure 4:**
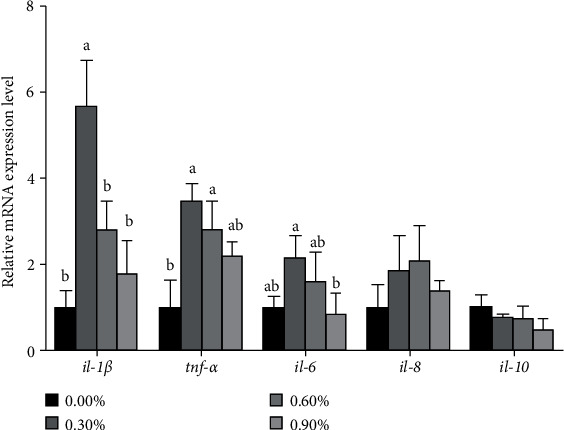
Effects of dietary CCD on *il-1β*, *tnf-α*, *il-6*, *il-8*, and *il-10* mRNA expression in a visceral mass. Vertical bars represent standard errors. There was no significant difference in bars bearing the same letters (*P* > 0.05, Tukey's test). *il-1β*: interleukin-1*β*; *tnf-α*: tumor necrosis factor-*α*; *il-6*: interleukin-6; *il-8*: interleukin-8; *il-10*: interleukin-10.

The activity of LZM increased with larvae fed with increasing concentration of CCD, and the significance was found in 0.90% group compared to the control group (*P* < 0.05) (Table 8). The activity of T-NOS and iNOS in larvae fed diet with 0.60% CCD was higher than the control group (*P* < 0.05) (Table 8). With the increase of chitosan concentration, the mRNA expression of *il-1β*, *tnf-α*, *il-6*, and *il-8* increased first and then decreased (Figure 4). Larvae fed the diet with 0.30% CCD had significantly higher *il-1β*, *tnf-α*, and *il-6* transcriptional levels than the control group (*P* < 0.05) (Figure 4). The expression of *il-8* and *il-10* had no significant difference among all treatments (*P* > 0.05) (Figure 4).

## 4. Discussion

The weak binding of MBD feeds could lead to deterioration of water quality, while the protective barrier on the surface of MCD was considered as a solution to reduce disassembly, inhibit oxidation, reduce nutrition loss, and improve water quality [10, 23]. In this experiment, the LEE and NRE significantly increased with appropriate concentration of chitosan (0.60~0.90% supplementation). Results indicated that CCD had better performance on LEE and NRE than the microencapsulated diet with ethyl cellulose (LEE: 85.3 ± 3.5%; NRE: 75.5 ± 4.7%) and gelatin (LEE: 76.8 ± 4.1%; NRE: 60.6 ± 5.2) [7]. The higher concentration of wall material correlated to the higher retention efficiency [24]. Previous study found that 85% of free amino acids from the gelatin microbound diet were leached into water compared to 17% from the protein-walled microencapsulated diet after 60 min [4]. Moreover, the leaching rate in this study was lower than microencapsulated delivery system of chitosan for giant freshwater prawn (*Macrobrachium rosenbergii*) larvae [9]. As a type of wall material, chitosan dramatically improved the stability of pellets in water and retained more water-soluble nutrients for the reason that chitosan is insoluble in water.

In addition to the excellent physical properties, this study also studied the special physiological functions of chitosan. The study demonstrated that larvae fed with 0.30~0.90% CCD had significant better growth performance than the control group, which was similar to previous studies in Nile tilapia (*Oreochromis niloticus*), cobia (*Rachycentron canadum*), and juvenile silver barb (*Barbonymus gonionotus*) [17, 25, 26]. Meanwhile, larvae fed with 0.90% CCD had lower final body weight than those fed with 0.60% CCD. Many experimental results have confirmed that the addition of high concentration chitosan was inconducive to the growth of animals [13, 17].

Digestive enzymes, including LAP and AKP, were considered as important indexes to evaluate the gut development and nutrition condition of larvae of aquatic animals [27–29]. In the present study, the activities of ɑ-Amylase (PS), trypsin (PS), AKP (BBM), and LAP (BBM) were significantly improved by 0.60% CCD, which may be related to that chitosan can restore intestinal microflora balance and improve intestinal mucosal barrier function [30]. 0.30% CCD could increase the expression of genes related to the development of larvae intestine, such as *zo-1*, *zo-2*, and *pcna*. The maturation of the larval intestine is closely related to the proliferation and differentiation of intestinal epithelial cells [31]. Previous studies showed that the height of microvilli of the distal intestine could increase, and the structural status of fish could be enhanced by dietary chitosan to have more osmoregulatory function [18, 32]. However, the results that larvae fed with 0.90% CCD had lower activity of lipase, and LAP demonstrated that high concentration of dietary chitosan supplementation could have slight negative effects on nutrient intake. The positive effects of CCD on the growth performance of larvae were probably due to the improvement of digestive enzyme activity and intestinal development to promote nutrient absorption.

High sensitivity to the environment could easily lead to oxidative stress in fish at an early stage, which was detrimental to growth and survival [33, 34]. Therefore, supplementation of antioxidants would be required. The study demonstrated that the larvae fed with 0.90% CCD had significantly higher activity of SOD than control group. SOD can catalytically eliminate superoxide radicals as a first-line defense mechanism against oxidative stress [35]. Furthermore, the content of GSH of large yellow croaker larvae fed with 0.30~0.60% CCD can be improved. GSH plays a pivotal role in cell resistance to oxidative and nitrosative damage through the way of eliminating potentially toxic oxidation products and reducing oxidized or nitrosated protein thiols [36]. MDA, as a biomarker of oxidative damage to lipids, were lower in larvae fed with chitosan than the control group, and this may be due to the increasing of the activity of SOD and the content of GSH. The results were replicated with giant tiger prawn (*Penaeus monodon*) and Nile tilapia [37, 38]. The antioxidant activity of chitosan and its derivatives has been clearly shown, but more authoritative reports need to be explored [39]. Overall, it could be inferred that chitosan was an excellent antioxidant to enhance antioxidant capacity to resist against oxidative damage of large yellow croaker larvae to promote its growth and survival.

Innate immunity offers a reasonable way to improve disease resistance at the early stage of larvae [40, 41]. Chitosan was verified as fishery immunostimulant in Nile tilapia and common carp (*Cyprinus carpio*) [25, 42]. In innate immunity system, LZM was involved in a broad battery of defense mechanisms, such as bacteriolysis and opsonization in fish [43]. In this study, larvae fed with 0.90% CCD significantly increased the activity of LZM. The same results were found in olive flounder (*Paralichthys olivaceus*) fed with chitosan-coated diets [10]. NO produced by iNOS has the functions of beneficial microbicidal, antiviral, and antiparasitic and then enhanced nitric oxide circulation mechanism in the body [44]. From the results of this experiment, larvae fed with 0.60% CCD had significantly higher activity of TNOS and subtype iNOS than control group. Furthermore, to investigate the positive effects of feeding CCD on the inflammatory response of large yellow croaker larvae, this study selected 5 genes related to immune defense and inflammatory reactions. Proinflammatory cytokines, including *il-1β*, *tnf-α*, and *il-6*, were commonly used immune regulatory genes in fish [43]. The upregulation of genes including *il-1β*, *tnf-α*, and *il-6* proved that chitosan could be used as an immunomodulator in large yellow croaker larvae. These cytokines above could enhance the immune response by promoting the proliferation, differentiation, and phagocytosis of immune cells, which might establish an early or timely protective immunity mechanism in fish species during challenge with multifarious pathogens [45–48]. These results provided a theoretical basis for chitosan as a fishery immunostimulant to promote growth and survival.

## 5. Conclusion

In summary, the results of the present study showed that chitosan-coated microdiets could significantly improve stability in water and retain more nutrients in the feed. Meanwhile, 0.30%~0.60% chitosan-coated microdiet could promote the growth performance and survival rate of large yellow croaker larvae probably through increasing digestive enzyme activity, antioxidant capacity, intestinal development, and inflammatory response. Chitosan as a biocompatible polymer can be considered as an appropriate wall material for preparing microcoated diets to deliver nutrients to large yellow croaker larvae.

## Figures and Tables

**Table 1:** Formulation and proximate analysis of the experimental diets (% dry matter).

Ingredient% dry diet	Experiment diets (concentration of chitosan used in wall material, *w*/*v*)			
	Control (0.00%)	Diet1 (0.30%)	Diet2 (0.60%)	Diet3 (0.90%)
White fish meal^1^	45.00	45.00	45.00	45.00
LT-krill meal^1^	20.00	20.00	20.00	20.00
Squid meal^1^	3.00	3.00	3.00	3.00
Yeast extract^1^	3.50	3.50	3.50	3.50
Vital wheat gluten^1^	5.00	5.00	5.00	5.00
Sodium alginate	2.00	2.00	2.00	2.00
*α*-Starch	3.00	3.00	3.00	3.00
Vitamin premix^2^	1.50	1.50	1.50	1.50
Mineral premix^2^	1.00	1.00	1.00	1.00
Monocalcium phosphate	2.00	2.00	2.00	2.00
Ascorbyl polyphosphate	0.20	0.20	0.20	0.20
Attractant mixture	2.00	2.00	2.00	2.00
Mould inhibitor	0.05	0.05	0.05	0.05
Antioxidant	0.05	0.05	0.05	0.05
Choline chloride	0.20	0.20	0.20	0.20
Fish oil	6.50	6.50	6.50	6.50
Astaxanthin	0.01	0.01	0.01	0.01
Soybean lecithin^1^	5.00	5.00	5.00	5.00
Analyzed nutrients composition (dry matter basis)				
Crude protein (%)	50.19	49.65	49.97	49.66
Crude lipid (%)	20.39	20.38	19.90	19.66

^1^Raw material was purchased from Qingdao Seven Good Biological Technology Co., Ltd, in Shandong, China; elementary composition (dry matter) referred to a previous study [31]. ^2^Vitamin premix (IU or g/kg), mineral premix (IU or g/kg): elementary composition referred to a previous study [31].

**Table 2:** PCR primer sequences used in this study.

Target gene	Forward primers (5′-3′)	Reverse primers (5′-3′)	Accession number
*zo-1*	TGTCAAGTCCCGCAAAAATG	CAACTTGCCCTTTGACCTCT	XM019260744
*zo-2*	ACCCGACCTGTTTGTTATTG	ATGCCGTGCTTGCTGTC	XM 027276911
*occludin*	AGGCTACGGCAACAGTTATG	GTGGGTCCACAAAGCAGTAA	XM010740442
*pcna*	GAGAGACAAGTGAGAGTTACCG	CTCTTTGTCTACATTGCTGGTCT	XM 010734227
*odc*	GAGCCAGGTCGCTTCTATG	CCGTGGTCCCTTCGTCT	XM 010736389
*tnf-α*	ACACCTCTCAGCCACAGGAT	CCGTGTCCCACTCCATAGTT	NM001303385
*il-1β*	CATAGGGATGGGGACAACGA	AGGGGACGGACACAAGGGTA	XM010736551
*il-6*	CGACACACCCACTATTTACAAC	TCCCATTTTCTGAACTGCCTCT	XM010734753
*il-8*	AATCTTCGTCGCCTCCATTGT	GAGGGATGATCTCCACCTTCG	XM010737667.3
*il-10*	AGTCGGTTACTTTCTGTGGTG	TGTATGACGCAATATGGTCTG	XM010738826
*β-Actin*	GACCTGACAGACTACCTCATG	AGTTGAAGGTGGTCTCGTGGA	GU584189

*il-1β*: interleukin-1*β*; *tnf-α*: tumor necrosis factor-*α*; *il-6*: interleukin-6; *il-8*: interleukin-8; *il-10*: interleukin-10; *zo-1*: tight junction protein-1; *zo-2*: tight junction protein-2; *pcna*: proliferating cell nuclear antigen; *odc*: ornithine decarboxylase.

**Table 3:** Setting velocity, LEE, and NRE of microcoated groups and the control group (Means ± S.E.M., *n* = 3).

Parameters	Experiment diets (concentration of chitosan used in wall material, *w*/*v*)				*P* value
	Control (0.00%)	Diet1 (0.30%)	Diet2 (0.60%)	Diet3 (0.90%)	
Setting velocity (cm/s)	2.20 ± 0.13	2.08 ± 0.11	2.03 ± 0.04	1.93 ± 0.08	0.355
LEE^1^ (%)	60.52 ± 1.40^b^	84.63 ± 2.03^a^	88.06 ± 1.67^a^	88.65 ± 1.37^a^	<0.001
NRE^1^ (%)	63.76 ± 3.82^b^	76.14 ± 2.07^ab^	79.52 ± 3.84^a^	84.68 ± 1.36^a^	0.006

Through Tukey's test, data with the same superscript letter in the same row have no significant difference (*P* > 0.05). ^1^LEE: lipid encapsulation efficiency; NRE: nitrogen retention efficiency.

**Table 4:** Effects of chitosan-coated diets on growth performance and survival rate (Means ± S.D., *n* = 3).

Parameters	Experiment diets (concentration of chitosan used in wall material, *w*/*v*)				*P* value
	Control (0.00%)	Diet1 (0.30%)	Diet2 (0.60%)	Diet3 (0.90%)	
IBW^1^ (mg)	3.81 ± 0.20	3.81 ± 0.20	3.81 ± 0.20	3.81 ± 0.20	—
FBW^1^ (mg)	75.33 ± 3.21^c^	127.00 ± 21.38^bc^	220.00 ± 6.08^a^	160.00 ± 36.50^b^	<0.001
SGR^1^ (%/day)	9.95 ± 0.14^c^	11.66 ± 0.59^b^	13.52 ± 0.09^a^	12.41 ± 0.72^ab^	<0.001
IBL^1^ (mm)	6.46 ± 0.41	6.46 ± 0.41	6.46 ± 0.41	6.46 ± 0.41	—
FBL^1^ (mm)	16.47 ± 0.56^c^	20.28 ± 0.64^b^	23.11 ± 0.57^a^	21.41 ± 1.03^ab^	<0.001
SR^1^ (%)	12.58 ± 0.26^b^	13.63 ± 0.50^ab^	14.73 ± 1.24^a^	13.70 ± 1.06^ab^	0.073

Through Tukey's test, data with the same superscript letter in the same row have no significant difference (*P* > 0.05). ^1^IBW: initial body weight; FBW: final body weight; SGR: specific growth rate; IBL: initial body length; FBL: final body length; SR: survival rate.

**Table 5:** Effects of chitosan-coated diets on body composition (Means ± S.E.M., *n* = 3).

Parameters	Experiment diets (concentration of chitosan used in wall material, *w*/*v*)				*P* value
	Control (0.00%)	Diet1 (0.30%)	Diet2 (0.60%)	Diet3 (0.90%)	
Crude protein (%)	56.11 ± 0.40	56.10 ± 0.34	55.58 ± 0.64	55.19 ± 1.01	0.712
Crude lipid (%)	20.49 ± 0.37	20.38 ± 0.28	19.90 ± 0.30	19.49 ± 0.05	0.094
Moisture (%)	87.31 ± 0.70	86.55 ± 1.69	85.25 ± 0.18	86.14 ± 0.61	0.534

Through Tukey's test, data with the same superscript letter in the same row have no significant difference (*P* > 0.05).

**Table 6:** Effects of chitosan-coated diets on the activities of amylase, lipase, and trypsin (Means ± S.E.M., *n* = 3).

Parameters		Experiment diets (concentration of chitosan used in wall material, *w*/*v*)				*P* value
		Control (0.00%)	Diet1 (0.30%)	Diet2 (0.60%)	Diet3 (0.90%)	
ɑ-Amylase (U/mg protein)	PS^1^	0.14 ± 0.02^b^	0.17 ± 0.01^b^	0.24 ± 0.03^a^	0.17 ± 0.00^b^	0.001
	IS^1^	0.16 ± 0.01^ab^	0.11 ± 0.01^c^	0.15 ± 0.01^b^	0.20 ± 0.01^a^	0.001
Trypsin (U/mg protein)	PS^1^	3.05 ± 0.43^b^	4.47 ± 0.34^a^	3.74 ± 0.07^ab^	3.53 ± 0.22^ab^	0.040
	IS^1^	3.50 ± 0.08	3.49 ± 0.14	3.45 ± 0.17	3.76 ± 0.14	0.709
Lipase (U/g protein)	PS^1^	0.49 ± 0.05	0.57 ± 0.07	0.41 ± 0.03	0.41 ± 0.04	0.133
	IS^1^	0.13 ± 0.01	0.14 ± 0.01	0.11 ± 0.01	0.10 ± 0.01	0.077
AKP^2^ (mU/mg protein)	BBM^2^	46.09 ± 4.00^b^	53.80 ± 2.69^b^	83.37 ± 1.01^a^	54.97 ± 3.13^b^	<0.001
LAP^2^ (mU/mg protein)	BBM^2^	4.77 ± 0.25^b^	5.02 ± 0.60^b^	7.29 ± 0.13^a^	3.13 ± 0.24^c^	<0.001

Through Tukey's test, data with the same superscript letter in the same row have no significant difference (*P* > 0.05). ^1^PS: pancreatic segments; IS: intestinal segments. ^2^AKP: alkaline-phosphatase; LAP: leucine-aminopeptidase; BBM: brush border membrane.

**Table 7:** Effects of chitosan-coated diets on the activities of antioxidant (Means ± S.E.M., *n* = 3).

Parameters	Experiment diets (concentration of chitosan used in wall material, *w*/*v*)				*P* value
	Control (0.00%)	Diet1 (0.30%)	Diet2 (0.60%)	Diet3 (0.90%)	
SOD^1^ (U/mg protein)	13.72 ± 1.33^b^	14.74 ± 2.29^b^	20.18 ± 2.54^ab^	27.27 ± 1.44^a^	0.005
T-AOC^1^ (Trolox/g protein)	0.13 ± 0.01^ab^	0.14 ± 0.00^a^	0.15 ± 0.00^a^	0.11 ± 0.00^b^	0.006
CAT^1^ (U/mg protein)	30.85 ± 4.09	42.43 ± 6.65	35.28 ± 6.76	31.85 ± 4.69	0.502
MDA^1^ (nmol/mg protein)	8.79 ± 0.42^b^	8.60 ± 0.25^b^	7.84 ± 0.52^ab^	6.79 ± 0.25^a^	0.021
GSH^1^ (nmol/mg protein)	55.58 ± 4.40^ab^	62.61 ± 6.32^a^	64.15 ± 3.85^a^	40.64 ± 1.62^b^	0.019
POD^1^ (U/mg protein)	1.72 ± 0.25	1.98 ± 0.16	2.40 ± 0.18	1.87 ± 0.32	0.280

Through Tukey's test, data with the same superscript letter in the same row have no significant difference (*P* > 0.05). ^1^SOD: superoxide dismutase; T-AOC: total antioxidant capacity; CAT: catalase; MDA: malondialdehyde; GSH: glutathione; POD: peroxidase.

**Table 8:** Effects of chitosan-coated diets on the activities of immunity (Means ± S.E.M., *n* = 3).

Parameters	Experiment diets (concentration of chitosan used in wall material, *w*/*v*)				*P* value
	Control (0.00%)	Diet1 (0.30%)	Diet2 (0.60%)	Diet3 (0.90%)	
LZM^1^ (U/mg protein)	93.60 ± 4.08^b^	108.31 ± 10.12^ab^	117.15 ± 17.62^ab^	163.06 ± 14.86^a^	0.024
TNOS^1^ (mU/mg protein)	2.05 ± 0.10^b^	2.31 ± 0.10^ab^	2.60 ± 0.16^a^	1.87 ± 0.03^b^	0.007
iNOS^1^ (mU/mg protein)	1.63 ± 0.06^ab^	1.91 ± 0.16^a^	2.01 ± 0.13^a^	1.36 ± 0.02^b^	0.014
cNOS^1^ (mU/mg protein)	0.42 ± 0.07	0.40 ± 0.07	0.60 ± 0.02	0.51 ± 0.04	0.227

Through Tukey's test, data with the same superscript letter in the same row have no significant difference (*P* > 0.05). ^1^LZM: lysozyme; TNOS: total nitric oxide synthase; iNOS: inducible nitric oxide synthase; cNOS: constitutive citric oxide synthase.

## Data Availability

The data that support the findings of this study are available from the corresponding author upon reasonable request.
